# Synthetic Double-Stranded RNAs Are Adjuvants for the Induction of T Helper 1 and Humoral Immune Responses to Human Papillomavirus in Rhesus Macaques

**DOI:** 10.1371/journal.ppat.1000373

**Published:** 2009-04-10

**Authors:** Christiane Stahl-Hennig, Martin Eisenblätter, Edith Jasny, Tamara Rzehak, Klara Tenner-Racz, Christine Trumpfheller, Andres M. Salazar, Klaus Überla, Karen Nieto, Jürgen Kleinschmidt, Reiner Schulte, Lutz Gissmann, Martin Müller, Anna Sacher, Paul Racz, Ralph M. Steinman, Mariagrazia Uguccioni, Ralf Ignatius

**Affiliations:** 1 Laboratory of Infection Models, German Primate Center, Göttingen, Germany; 2 Institute of Microbiology and Hygiene, Department of Infection Immunology, Charité–University Medicine Berlin, Campus Benjamin Franklin, Hindenburgdamm, Berlin, Germany; 3 Institute for Research in Biomedicine, Bellinzona, Switzerland; 4 Bernhard Nocht Institute for Tropical Medicine, Hamburg, Germany; 5 Laboratory of Cellular Physiology and Immunology, The Rockefeller University, New York, New York, United States of America; 6 Oncovir Inc., Washington, D.C., United States of America; 7 Department of Molecular and Medical Virology, Ruhr-University Bochum, Bochum, Germany; 8 Infection and Cancer Research Program, German Cancer Research Center (DKFZ), Heidelberg, Germany; 9 Department of Botany and Microbiology, King Saud University, Riyadh, Saudi Arabia; National Cancer Institute, United States of America

## Abstract

Toll-like receptor (TLR) ligands are being considered as adjuvants for the induction of antigen-specific immune responses, as in the design of vaccines. Polyriboinosinic-polyribocytoidylic acid (poly I:C), a synthetic double-stranded RNA (dsRNA), is recognized by TLR3 and other intracellular receptors. Poly ICLC is a poly I:C analogue, which has been stabilized against the serum nucleases that are present in the plasma of primates. Poly I:C_12_U, another analogue, is less toxic but also less stable in vivo than poly I:C, and TLR3 is essential for its recognition. To study the effects of these compounds on the induction of protein-specific immune responses in an animal model relevant to humans, rhesus macaques were immunized subcutaneously (s.c.) with keyhole limpet hemocyanin (KLH) or human papillomavirus (HPV)16 capsomeres with or without dsRNA or a control adjuvant, the TLR9 ligand CpG-C. All dsRNA compounds served as adjuvants for KLH-specific cellular immune responses, with the highest proliferative responses being observed with 2 mg/animal poly ICLC (p = 0.002) or 6 mg/animal poly I:C_12_U (p = 0.001) when compared with immunization with KLH alone. Notably, poly ICLC—but not CpG-C given at the same dose—also helped to induce HPV16-specific Th1 immune responses while both adjuvants supported the induction of strong anti-HPV16 L1 antibody responses as determined by ELISA and neutralization assay. In contrast, control animals injected with HPV16 capsomeres alone did not develop substantial HPV16-specific immune responses. Injection of dsRNA led to increased numbers of cells producing the T cell–activating chemokines CXCL9 and CXCL10 as detected by in situ hybridization in draining lymph nodes 18 hours after injections, and to increased serum levels of CXCL10 (p = 0.01). This was paralleled by the reduced production of the homeostatic T cell–attracting chemokine CCL21. Thus, synthetic dsRNAs induce an innate chemokine response and act as adjuvants for virus-specific Th1 and humoral immune responses in nonhuman primates.

## Author Summary

Novel adjuvants that facilitate the induction of strong cellular immunity could be of help in the design of vaccine strategies to combat infections such as HIV or tuberculosis. Our immune cells possess archaic receptors recognizing structures of infectious pathogens, and the interaction of these receptors with their ligands results in an activation of the immune system. Here we exploited synthetic forms of one of these ligands, i.e., dsRNA, to define an adjuvant for the induction of cellular immune responses in primates. We injected model and viral proteins together with three different forms of dsRNA subcutaneously (s.c.) in rhesus macaques, and all compounds served as adjuvants for the induction of cellular immunity without the incidence of major side effects. These adjuvant effects depended on the adjuvant dose and coincided with profound alterations in the chemokine production in the draining lymph nodes. dsRNA also helped to induce cellular and humoral immune responses against capsomeres of low immunogenicity derived from the human papillomavirus 16, the causative agent in about 50% of all cases of cervical cancer worldwide. Therefore, formulations involving synthetic dsRNA are promising candidates for development of novel vaccines.

## Introduction

Effective vaccines against infections caused by intracellular pathogens including HIV infection, malaria, or tuberculosis most likely will need to induce strong cellular and humoral immune responses [1]. Current vaccine strategies under development are based on prime-boost immunizations, such as vaccination with plasmid DNA followed by booster injections with replication-incompetent viral vectors (e.g., adenoviruses or poxviruses), with both DNA and viruses encoding immunogenic proteins of the pathogen [2]. There is concern that these strategies may be insufficiently immunogenic and protective, so alternative vaccine approaches are under development [3],[4]. While protein based vaccines allow the delivery of large amounts of immunogenic vaccine antigens, particularly when targeted to antigen presenting dendritic cells (DCs) [5], these vaccines require the identification of appropriate adjuvants [6], which may act by differentiating the DCs to elicit strong immunity [7]–[10]. Monkeys are being used as an animal model to develop AIDS vaccines and are likely to be a valuable preclinical model to identify adjuvants and understand their mode of action.

Currently the most widely used adjuvant is aluminum hydroxide. It predominantly induces Th2 immune responses [11], and as such may be inappropriate for HIV or tuberculosis vaccines or for immune therapy of tumors related to infection by human papillomaviruses (HPV). Ligands for pathogen recognition receptors, e.g., Toll-like receptor (TLR) ligands, can stimulate cells of the innate and adaptive immune systems and have therefore been proposed as promising adjuvant candidates [12],[13]. We have previously studied the effects of TLR9 ligands, i.e., CpG-A and CpG-B, on the induction of protein-specific immune responses in nonhuman primates. However, we did not observe strong CD4^+^ T cell-mediated immune responses as indicated by T cell proliferative assays [14]. This may in part be due to the lack of TLR9 expression in myeloid primate DCs [15], which can be valuable for the priming of naïve T cells and the induction of cellular immune responses [16],[17]. In this study, we have focused on synthetic double stranded RNA (dsRNA) compounds as adjuvants. They can be recognized by both TLR3 [18] and the melanoma differentiation-associated gene-5 (MDA-5) [19], pattern recognition receptors that are expressed by many cell types and are involved in anti-viral immune responses [20].

In mice, polyriboinosinic-polyribocytoidylic acid (poly I:C) has long been known as a strong IFN-α inducer and provides anti-viral and adjuvant activity [21],[22]. Poly I:C also works as a mucosal adjuvant for the induction of humoral and cell-mediated immune responses [23]–[25]. MDA-5 is important for the IFN response induced by poly I:C [26],[27].

In primates, poly I:C is a less effective IFN-α inducer, most likely due to nucleases, which reduce the biostability of poly I:C and are reported to be more prevalent in the serum of primates than rodents [28]. A complex of poly I:C with poly-L-lysine and carboxymethylcellulose (poly ICLC), however, is five to 10 times more resistant to hydrolysis by RNAse in primate serum than the parent poly I:C and induces significant levels of interferon in monkeys under conditions in which poly I:C itself induces no interferon [29],[30]. Poly ICLC possesses anti-viral activity against a variety of viruses in monkeys [31]–[33] and chimpanzees [34], and also inhibits malaria infection of macaques [35]. Furthermore, it has shown potent adjuvant activity on the induction of humoral immune responses in the nonhuman primate models of Venezuelan equine encephalomyelitis virus and swine influenza virus [36],[37]. In humans, dose-dependently, mild to moderate side effects of poly ICLC were observed in a number of phase I and II studies conducted in children and adults [38]–[45]. Another synthetic dsRNA, poly I:C_12_U (Ampligen), supports the induction of broad antiviral immune responses in mice [46],[47], shows low toxicity in humans [48], and should therefore also be considered as an adjuvant in human vaccine trials. To date, no studies have been reported on the potential of synthetic dsRNA to augment cellular immunity in primates.

We therefore have performed studies in rhesus macaques to address the impact of dsRNA on the induction of protein-specific immune responses. As a prelude to studies with protein based vaccines, we selected keyhole limpet hemocyanin (KLH). In contrast to a previous study where TLR7/8 and TLR9 ligands have been used as adjuvants for cellular immunity in rhesus macaques [49], we injected the dsRNA plus KLH in aqueous solution without additional emulsification in water-in-oil adjuvants, such as Montanide, to minimize the risk of undesired side-effects at the site of injection. To confirm that the adjuvant effect of dsRNA is also manifest in the context of the injection of viral proteins, we injected some animals with the major capsid protein (L1) of HPV16 with or without poly ICLC. HPV16 is the major carcinogenic genotype of HPV in most countries and involved in about 50% of the cases of cervical cancer worldwide [50]. Recently, prophylactic vaccines against HPV16 have been marketed that consist of L1 virus-like particles (VLPs) and induce neutralizing antibodies that efficiently protect against persistent HPV infection and premalignant cervical lesions [51]. However, therapeutic vaccines for the use in individuals who are already infected will need to induce cellular immunity, most likely against the E6/E7 antigens of HPV. Subunits of VLPs (pentameric capsomeres) have potential advantages over VLPs, i.e., higher stability and reduced production costs but their immunogenicity has not yet been evaluated in nonhuman primates.

To monitor also the innate response to dsRNAs, we concentrated on the rapid innate production of CXCL9 (MIG) and CXCL10 (IP-10) chemokines, which are induced by dsRNA [52] as well as CCL21 (SLC), which attracts naïve T lymphocytes and DCs [53]. Here we show that dsRNAs act as adjuvants for the induction of innate and adaptive cellular and humoral immunity in nonhuman primates.

## Results

### Synthetic dsRNAs are effective adjuvants for the induction of protein-specific cellular immune responses

Poly ICLC has adjuvant activity on the induction of humoral immunity at doses as low as 0.1 mg/kg [37]. Since we assumed that higher doses might be required for the induction of cellular immune responses, we immunized rhesus macaques subcutaneously (s.c.) with KLH and either poly ICLC (0.5 mg/kg body weight; 6 animals), poly I:C (0.5 mg/kg; 4 animals), or without adjuvant (4 animals). To monitor the development of T cell immunity, we cultured peripheral blood mononuclear cells (PBMC) with or without KLH and determined whether immunization resulted in T cell proliferative responses to the administered antigen by ^3^H thymidine incorporation assays. Peak stimulation indices (SI; KLH-induced proliferation divided by proliferation in medium alone) were significantly higher (p = 0.040) in animals injected with poly ICLC (week 0, 1.93±1.38; peak, 23.00±8.02) compared with KLH alone (week 0, 3.28±1.55; peak, 8.97±7.47), individual maximum proliferative responses are shown in Table S1. The kinetics of the responses are shown in the Figure S1 and reveal significantly stronger proliferative responses in poly I:C co-injected animals than in controls six weeks post injection (p = 0.03). Thus, KLH-dependent proliferation of PBMCs was induced with poly ICLC or poly I:C by the s.c. route.

**Figure 1:**
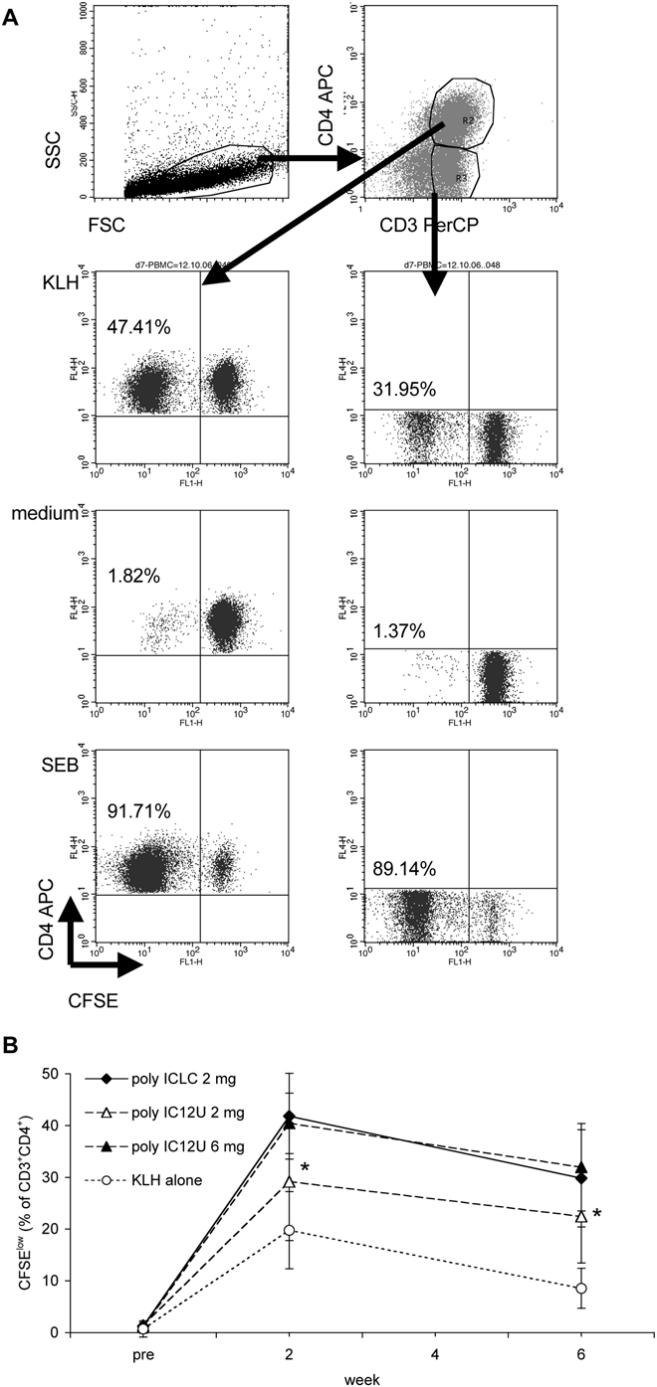
Induction of KLH-specific proliferation by immunization with KLH plus poly ICLC or poly I:C_12_U. (A) Live cells in a T cell gate (SSC^low^ cells) were analyzed using anti-CD3 PerCP- and anti-CD4 or anti-CD8 APC-conjugated mAbs. CD3^+^CD4^+^ and CD3^+^CD4^−^, or CD3^+^CD8^+^ and CD3^+^CD8^−^ cells, respectively, were further analyzed regarding their CFSE expression. The indicated percentages are calculated relative to the number of gated cells. (B) KLH (200 µg) was administered either alone or in combination with 2 mg poly ICLC, 2 mg poly I:C_12_U, or 6 mg poly I:C_12_U per animal (five animals per group). CFSE-dilution was used to assess KLH-specific proliferation of CD3^+^CD4^+^ T cells before as well as 2 and 6 weeks after immunization. Background proliferation (percentage of CFSE^low^ cells in medium alone) was subtracted from the percentage of CFSE^low^ cells after stimulation with 100 µg/ml KLH for 6 days, and means±SD for each group are presented. * Statistical differences at 2 weeks: 6 mg poly I:C_12_U versus KLH alone (p = 0.001), 2 mg poly ICLC versus KLH alone (p = 0.002), 2 mg poly I:C_12_U versus KLH alone (p = 0.16); at 6 weeks: 2 mg poly I:C_12_U versus KLH alone (p = 0.013).

The poly I:C analogue poly I:C_12_U requires TLR3 to be active in vivo [54],[55] and shows little toxicity in humans [48]. Like poly I:C and in contrast to poly ICLC, it is not stabilized against primate serum nucleases. We therefore compared the effectiveness of poly I:C_12_U to poly ICLC in a separate study, using fixed standardized doses per animal rather than adjustment to body weight. To study the effects of dsRNA on cellular immune responses in more detail, we used a carboxyfluorescein diacetate succinimidyl ester (CFSE) dilution assay, which allows the separate evaluation of CD4^+^ and CD8^+^ T cells (Figure 1A). KLH at 200 µg/animal was administered to five animals each either alone or together with 2 or 6 mg poly I:C_12_U or 2 mg poly ICLC per animal (i.e., 0.27 to 0.44, 1.00 to 1.13, and 0.33 to 0.43 mg/kg, respectively). The dose of 2 mg per animal has been used in previous studies on TLR agonists as vaccine adjuvants in monkeys [49] and thus facilitates comparison between studies of different adjuvant compounds. KLH-specific CD4^+^ T-cell proliferation at week 2 after immunization was significantly higher when KLH was given with either 6 mg poly I:C_12_U (p = 0.001) or 2 mg poly ICLC (p = 0.002) whereas at that time point no significant difference to KLH alone was observed after the injection of 2 mg poly I:C_12_U (p = 0.16; Figure 1B). The effect of immunization with poly I:C_12_U or poly ICLC on proliferative responses was sustained over 6 weeks, and there was a significant difference also for the 2 mg poly I:C_12_U group over KLH alone at this time point (p = 0.013; Figure 1B). Thus, all three synthetic dsRNA compounds that we tested could serve as adjuvants for the induction of protein-specific T-cell proliferation in primates.

To examine possible dose-dependent adjuvant effects of poly ICLC, we compared in a prime-boost experiment the effects of 0.5 mg/kg body weight with those of 0.1 mg/kg body weight, which is still sufficient for the induction of humoral immune responses [37]. After the first immunization increased KLH-specific CD3^+^CD4^+^ T cell proliferative responses were seen in both animals immunized with 0.5 mg poly ICLC/kg, and CD3^+^CD4^−^ T cells (as a surrogate for CD8^+^ T cells) were expanded to a similar extent (Fig. S2A). CD4^+^ and CD4^−^ T cell-proliferative responses were less pronounced after the primary immunization together with the lower 0.1 mg/kg dose of poly ICLC (Figure S2B). Booster immunization at week 14 enhanced the proliferative CD4^+^ T cell responses in the animal 13404 immunized with 0.5 mg poly ICLC/kg (Figure S2A) and in the animal 13406 receiving the lower dose of poly ICLC (Figure S2B). Therefore, 0.5 mg/kg of poly ICLC might be more active as an adjuvant for cellular immunity than lower doses.

### Injection of poly ICLC supports the induction of HPV-specific Th1 and humoral immune responses

To confirm that dsRNA analogues also serve as adjuvants in the context of a clinically relevant viral antigen, we injected s.c. six animals each with a low dose of HPV16 L1 capsomeres (10 µg) with or without 2 mg of poly ICLC. Another six animals were injected with 2 mg of the TLR9 ligand CpG-C (ODN 2396), which supports the induction of protein-specific cellular immune responses in monkeys when injected in a water-in-oil emulsion [49]. We selected the L1 pentamers rather than the complete virus-like particles (VLP; 360 molecules of L1), since capsomeres are promising candidates for 2^nd^ generation vaccines but their immunogenicity in nonhuman primates has not yet been evaluated. The capsomeres were obtained by expression of a modified L1 protein in baculovirus-infected insect cells [56]. In the immune assays, we re-stimulated PBMCs with HPV16 VLPs and used mouse norovirus VLPs (A447) generated in the same expression system as a negative control antigen. All animals were boosted with a second injection of antigen +/− adjuvant eight weeks later.

**Figure 2:**
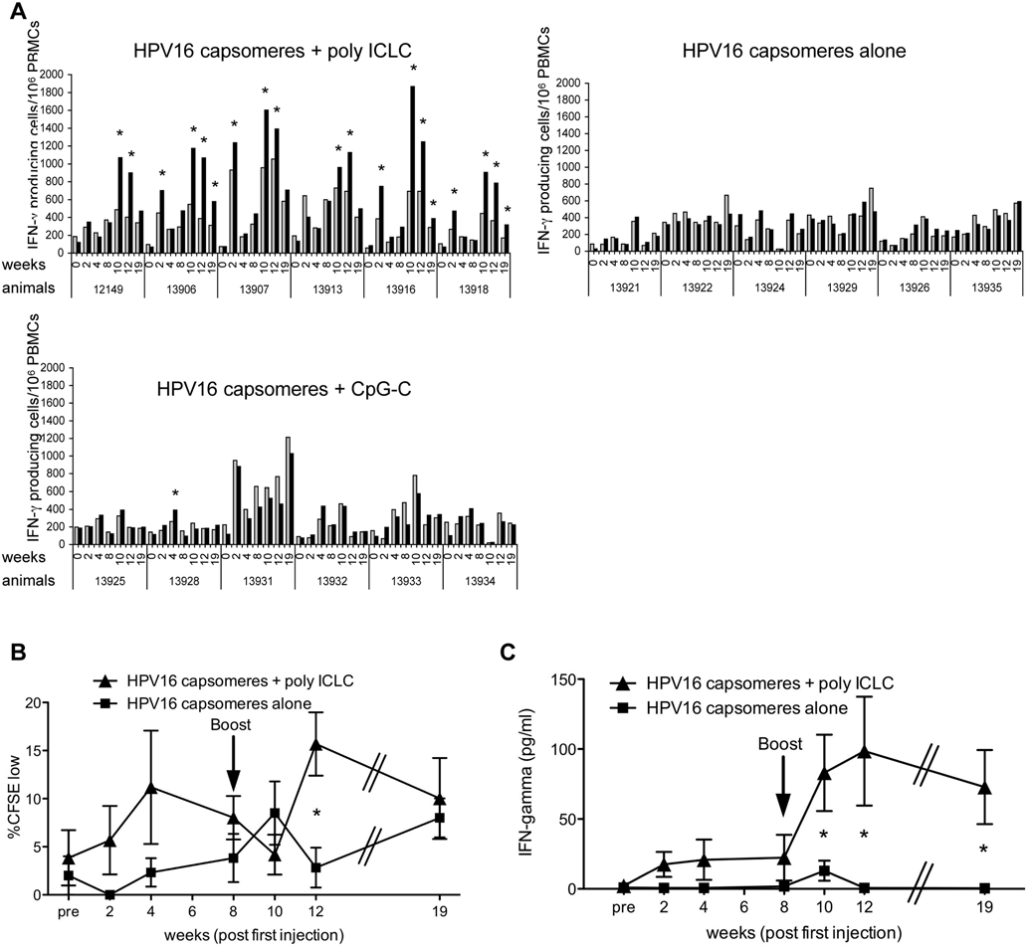
Induction of HPV-specific Th1 immune responses by injection of HPV16 L1 protein and poly ICLC. HPV16 capsomeres (10 µg/animal) were injected either alone or in combination with 2 mg poly ICLC or 2 mg CpG-C per animal at weeks 0 and 8 (six animals per group), and immune responses were monitored in triplicates in IFN-γ ELISPOT assays (A), CFSE dilution assays gated on CD4^+^ T cells (B), and IFN-γ concentrations in supernatants of re-stimulated PBMCs (C), at baseline (pre) and the indicated points in time after the first immunizations. At week 19, only five of the six poly ICLC-injected animals could be analyzed for proliferation and IFN-γ secretion. (A) To determine the numbers of HPV-specific IFN-γ secreting cells, PBMCs were re-stimulated with HPV or control antigen (A447) at 1.25 µg/ml for 20 hours, the ELISPOT plates developed, and the spot numbers counted and extrapolated to 10^6^ PBMCs (HPV16, black bars; A447, light grey bars). Average spot numbers of background responses (to A447) plus twice the standard deviation were considered positive (*). (B) HPV-specific proliferative responses were determined using CFSE-labeled PBMCs expanded for 6 days in the presence of HPV16 or control antigen (A447) at 1.25 µg/ml and re-stimulated with peptide pools 1–4 (B) or 5–8 (data not shown) for the final 6 hours of the assay. Data shown as mean %CFSE^low^ (HPV16) minus background proliferation (%CFSE^low^ with A447)±SEM. * p = 0.008. (C) PBMCs were re-stimulated with HPV16 or control antigen (A447) at 1.25 µg/ml for 2 days, supernatants were collected, and IFN-γ concentrations determined using a monkey IFN-γ specific ELISA assay. Data shown as mean IFN-γ secretion (pg/ml) by cells re-stimulated with HPV16 minus background secretion (by cells incubated in the presence of A447)±SEM. * p = 0.033 at week 10, p = 0.031 at week 12, and p = 0.014 at week 19.

Numbers of IFN-γ secreting cells in the peripheral blood were determined by ELISPOT assay. At week 2, we detected increased numbers of HPV-specific, IFN-γ secreting cells in PBMCs from 4 out of 6 animals (Figure 2A) injected with antigen plus poly ICLC, and the responses waned in all animals by week four. Two weeks after the booster injection (at week 10 after the first injections), however, all six animals injected with antigen together with poly ICLC had developed HPV-specific, IFN-γ secreting cells, which also were maintained two weeks later (week 12) and still present in 3 animals at week 19. In contrast, IFN-γ secreting cells were detectable at elevated numbers in only one of the CpG-injected monkeys (animal 13928) four weeks after the first injection and this response could not be boosted by the second injection. None of the control animals showed substantial numbers of HPV-specific IFN-γ secreting cells, neither following the first nor the booster injection (Figure 2A). The background responses against A447 might be induced by contaminating protein fractions derived from the expression system, in which both antigens, i.e., HSV16 L1 capsomeres and A447, had been generated.

When we assessed T cell proliferation in CFSE assays, we found significantly enhanced HPV-specific CD4^+^ T-cell proliferative responses in the poly ICLC-injected monkeys four weeks after the second application of antigen (p = 0.008) (Figure 2B). Figure 2B depicts the proliferation of CD3^+^CD8^−^ cells, re-stimulated for the last 6 h of the assay with peptide pools 1–4. Similar results (p = 0.012, week 12) were obtained with cells re-stimulated for the final 6 h with pools 5–8 (data not shown). At week 19, proliferative responses did not differ significantly in poly ICLC-injected and control animals.

To further characterize the Th cell responses, we determined the concentrations of IFN-γ, IL-4, and IL-17 in supernatants collected from re-stimulated PBMCs 2 d after setting up the assays. We used ELISAs for the detection of monkey cytokines or, in the case of IL-17 an ELISA for the detection of the human protein but known to cross-react with monkey IL-17 [57]. Following the booster injection at week 8, we detected significantly more IFN-γ in the supernatants of cells collected from poly ICLC-injected animals than in those of cells from control animals and these responses were sustained until week 19 (Figure 2C). In contrast, we were unable to detect IL-4 or IL-17 in the supernatants from assays set up with PBMCs from either group of animals. Thus, poly ICLC supports the induction of HPV-specific Th1 immune responses, i.e., CD4^+^ T cell proliferative responses and IFN-γ secretion.

**Figure 3:**
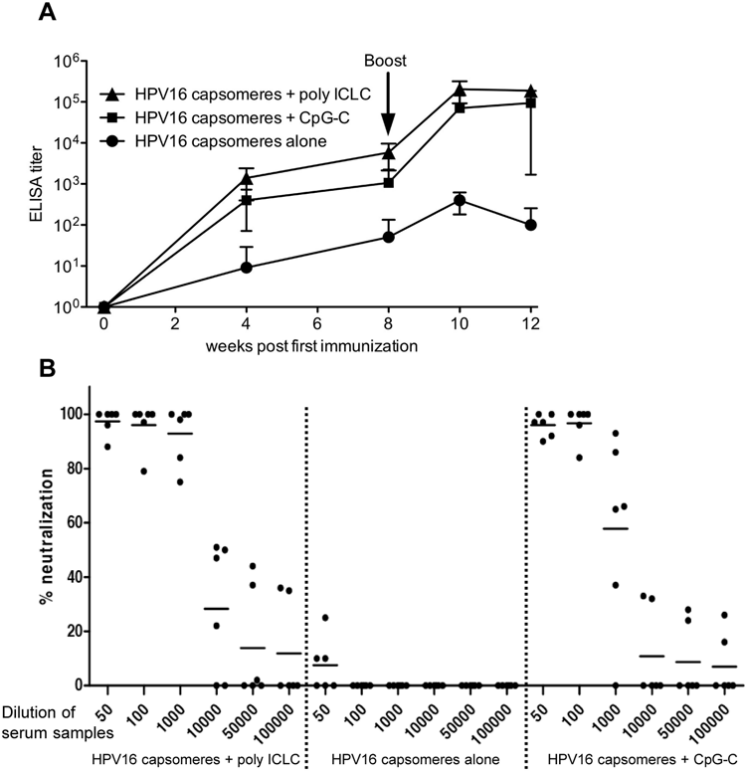
Both poly ICLC and CpG-C act as adjuvants for the induction of HPV16-specific binding and neutralizing antibodies. HPV16 capsomeres (10 µg/animal) were injected either alone or in combination with 2 mg poly ICLC or 2 mg CpG-C per animal at weeks 0 and 8 (six animals per group), and L1-specific antibodies in plasma samples were measured (A) by ELISA at baseline (pre) and the indicated points in time after the first immunization, and (B) by neutralization assays at week 12. Data given as mean titers +/− SD (A), and as the percentage of neutralization of pseudovirions by serum samples at the dilutions indicated [(B), line, median; *, individual animals)], measured by reduction of SEAP activity (see Materials and Methods) and compared to controls of untreated pseudovirions.

We also determined the humoral immune responses induced by the injection of HPV16 L1 capsomeres with or without adjuvants. Injection of poly ICLC or CpG-C resulted in up to 1000fold increased titers of binding antibodies (measured by ELISA) compared with control animals (Figure 3A; p<0.01 for both adjuvants for weeks 4, 8, 10, and 12), and at weeks 4, 8, and 10, poly ICLC also induced higher titers than an equal dose of CpG-C (p<0.05 for week 4, p<0.01 for weeks 8 and 10). The individual antibody titers of all animals are shown for all points in time in the Table S2. In addition, we performed neutralization assays using the serum samples collected 12 weeks after first immunization and HPV16 pseudovirions as targets. Sera of the animals from both adjuvant groups showed considerable neutralizing activity while samples from the control animals were not able to neutralize the activity of the pseudovirions in our assay (Figure 3B). Poly ICLC injected animals showed stronger responses than monkeys that had received CpG-C (p = 0.03 for serum dilutions of 1∶1000). There was a good correlation between ELISA and neutralization titers in the sera of the individual animals (Figure S3). Therefore, while CpG-C mainly affects the induction of antibodies, poly ICLC acts as adjuvant for both humoral and cellular immunity.

### Injection of KLH plus synthetic dsRNA induces an innate chemokine response in the T cell areas of draining lymph nodes and serum

Since we have previously observed that poly I:C activates monkey DCs [58], immunohistochemistry was performed to determine the number and activation status of DCs present in lymph nodes taken prior to immunization and at 18 h after injection of poly ICLC. The numbers of phenotypically immature (CD1a^+^) and mature (CD83^+^ or CD208^+^) DCs varied between animals but did not show a clear decrease or increase after immunization (Figure S4).

**Figure 4:**
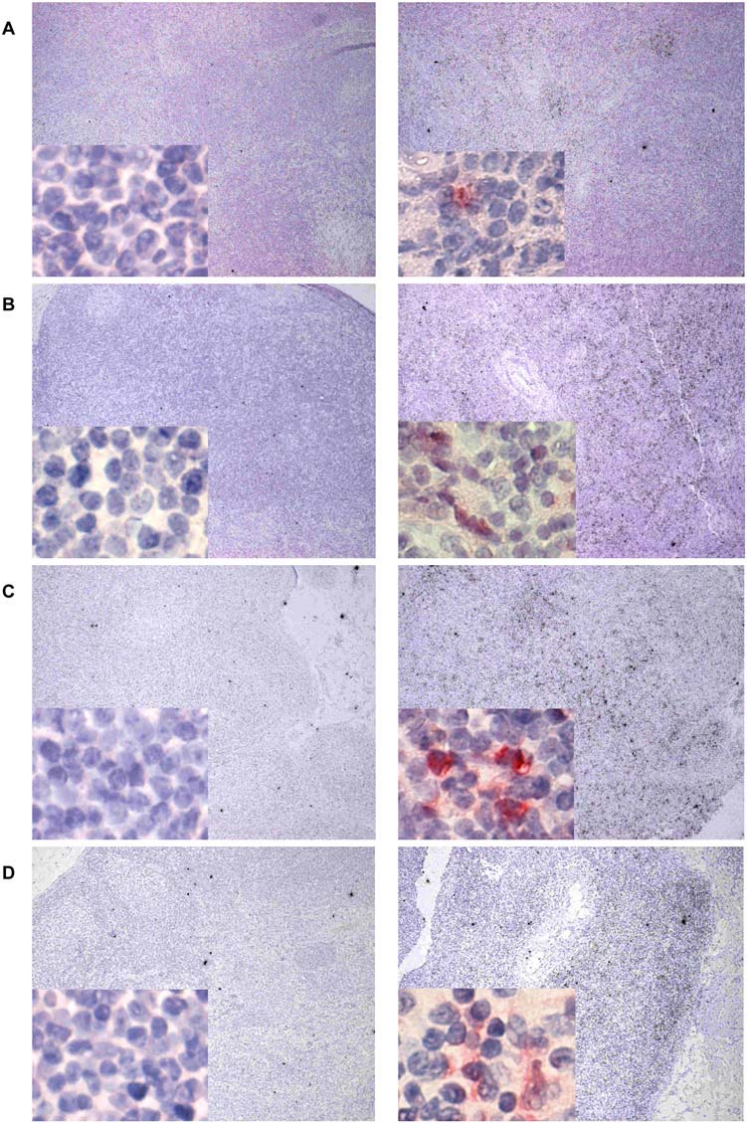
Upregulation of CXCL10 and CXCL9 in draining lymph nodes following injection of poly ICLC. Expression of CXCL10 (A,B) and CXCL9 (C,D) mRNA (in situ hybridization) and protein (insets) in control lymph nodes before (left panels) and in draining lymph nodes (right panels) 18 hours after KLH-immunization with 0.5 (A,C) or 0.1 mg/kg (B,D) poly ICLC, respectively. The presence of chemokine mRNA is shown by the black dots in the T cell area (100× magnification). Cells positive for chemokine protein expression are visible in red (insets: 400× magnification). Immunohistochemistry was performed on sections consecutive to the one in which in situ hybridization was performed to confirm protein expression. Data are representative for two animals for each dose of poly ICLC.

**Figure 5:**
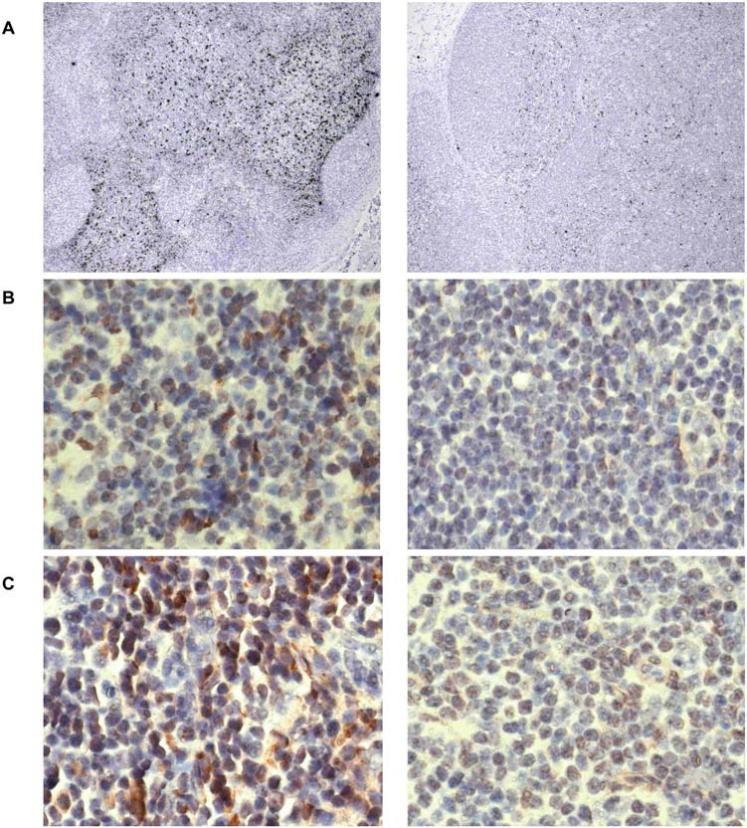
Downregulation of CCL21 in draining lymph nodes 18 hours after immunization with KLH plus poly ICLC. (A) Expression of CCL21 mRNA (determined by in situ hybridization) in control lymph nodes before (black dots, left panel) and in draining lymph nodes (right panel) 18 hours after KLH-immunization with 0.5 mg/kg poly ICLC; in situ hybridization, 100× magnification. (B,C) Cells positive for CCL21 protein expression (determined by immunohistochemistry) are visible in brown in the T cell areas of control lymph nodes (left panels) and of draining lymph nodes (right panels) after immunization with KLH plus 0.5 mg/kg (B) or 0.1 mg/kg (C) poly ICLC; 400× magnification.

Draining inguinal lymph nodes were also analyzed for CXCL10, CXCL9, and CCL21 by in-situ hybridization and immunohistochemistry. In comparison to control lymph nodes removed before immunizations, elevated expression of CXCL10 (Figure 4A and 4B) and CXCL9 (Figure 4C and 4D) was detected in the T cell areas of draining lymph nodes at 18 hours after immunization. Chemokine mRNA expression correlated with protein expression detected by immunohistochemistry (insets in Figure 4). Expression of CCL21 mRNA (Figure 5A) and protein (Figure 5B and 5C) 18 hours after immunization was markedly decreased in draining lymph nodes compared with control lymph nodes obtained before immunization. Thus, the innate response to dsRNA is detectable in lymph node cells.

**Figure 6:**
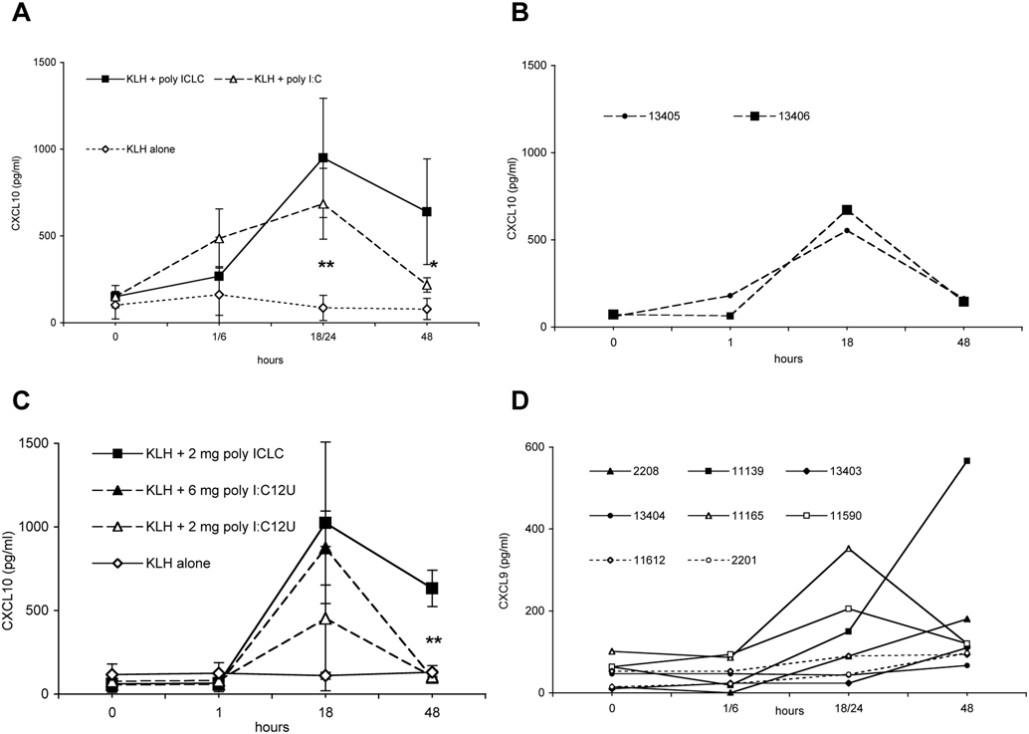
dsRNA-induced CXCL10 and CXCL9 in serum. (A) Mean serum levels (±SD) of CXCL10 after immunization of animals with either KLH alone (n = 4), KLH+0.5 mg/kg poly I:C (n = 4 animals), or KLH+0.5 mg/kg poly ICLC (n = 6 animals). (B) The effect of lower doses of poly ICLC on CXCL10 serum levels was assessed in two animals immunized with 0.1 mg/kg. (C) Mean serum levels (±SD) of CXCL10 after immunization of five animals per group with either KLH alone, KLH+6 mg poly IC_12_U, KLH+2 mg poly IC_12_U, or KLH+2 mg poly ICLC. (D) Serum levels of CXCL9 after immunization with either KLH alone (n = 2, dotted lines), KLH+0.5 mg/kg poly I:C (full lines, open symbols), or KLH+0.5 mg/kg poly ICLC (full lines, full symbols). Chemokine levels were determined at the indicated points in time; *, p = 0.027; **, p≤0.001.

The administration of poly ICLC or poly I:C together with KLH led to a significant increase of serum levels of CXCL10 (Figure 6A; p = 0.001 for both compounds at 18 or 24 h). Furthermore, 48 h after immunization, serum levels of CXCL10 were significantly higher in poly ICLC- than in poly I:C-injected monkeys (p = 0.027). Like poly I:C at 0.5 mg/kg, poly I:C_12_U or lower doses of poly ICLC (0.1 mg/kg) induced increased CXCL10 levels, which were less sustained (Figure 6B and 6C).

We detected increased CXCL9 serum levels in animals injected with poly I:C or poly ICLC (0.5 mg/kg), and there were minor changes in CXCL9 concentrations in monkeys receiving KLH alone (Figure 6D). No changes of CXCL9 serum concentrations were observed when 0.1 mg/kg poly ICLC were administered (data not shown). At 6, 24, or 48 h after infection, no significant differences in serum levels of IFN-α, IFN-γ, TNF, IL-12p40, and CCL3 (MIP-1α) were observed between groups receiving KLH alone or together with dsRNA (data not shown). We were not able to detect considerable serum concentrations of IFN-α at any point in time including 1 h post injection.

### CXCL10 production by rhesus macaque DCs activated through dsRNA

**Figure 7:**
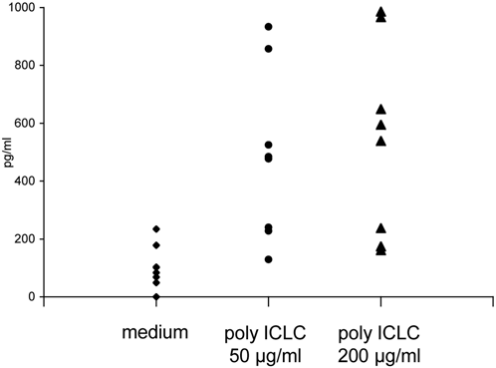
Monkey DCs secrete CXCL10 upon stimulation with poly ICLC. Monocyte-derived DCs were generated from rhesus macaque CD14^+^ monocytes and either stimulated with poly ICLC at the indicated concentrations or left in medium alone. CXCL10 concentrations in the supernatants collected 48 hours later were determined by ELISA. Results of seven independent experiments are given, the horizontal lines indicate the mean concentrations.

Since immunohistochemistry and in-situ hybridization revealed that CXCL10 was mainly produced in the T cell-areas of the draining lymph nodes (Figure 4), we considered DCs as a potential source for this chemokine in vivo. Unfortunately, double-labeling with DC identifying mAbs was not possible on formalin-fixed specimens. We therefore tested whether dsRNA may directly induce CXCL10 secretion by highly purified rhesus macaque DCs in vitro. When monocyte-derived monkey DCs were incubated with poly ICLC at two different concentrations (50 and 200 µg/ml), significantly elevated CXCL10 concentrations were detectable 48 h later in the cell culture supernatants (p = 0.002 compared to un-stimulated controls), and both doses of of poly ICLC induced comparable levels of CXCL10 (Figure 7). Thus, primate DCs produce CXCL10 upon stimulation with synthetic dsRNA, making DCs one of the candidate sources of CXCL10 observed in the draining lymph nodes.

## Discussion

This study shows that s.c. injection of synthetic dsRNA, i.e., poly I:C, poly ICLC, or poly I:C_12_U supports the induction of cellular immune responses to protein antigens in nonhuman primates. These responses could also be boosted by a second injection of antigen together with dsRNA. We observed antigen-specific T cell proliferation of CD3^+^CD4^+^ and CD3^+^CD4^−^ T cells. High but nontoxic doses (toxicity starts in *M. mulatta* at i.v. doses >2 mg/kg, i.m. or s.c. injections are better tolerated than i.v. injections; unpublished observations) of poly ICLC (0.5 mg/kg or 2 mg/animal) might be more potent than lower doses (≤0.1 mg/kg). Using HPV16 capsomeres at low doses (10 µg/animal) as a relevant viral antigen with low immunogenicity, we also showed that poly ICLC, but not CpG-C (which supported the induction of humoral responses, however), supports the induction of HPV16-specific Th1 responses. The lack of effect of CpG-C in our system compared to other studies where the same compound helped to elicit cellular immunity in nonhuman primates is most likely due to the fact that we injected the antigens in PBS, while others injected CpG-C and antigens in the synthetic water-in-oil emulsion, Montanide [49]. Amongst the three different formulations of synthetic dsRNA, poly ICLC appears to possess the most potent adjuvant activity on the induction of cellular immune responses. Subsequent studies will show whether it will help to induce protective immune responses against other pathogens, e.g., SIV.

Both adjuvants supported the induction of humoral immune responses, including neutralizing antibodies. Therefore, subsequent in vivo studies should compare poly ICLC with the adjuvants currently used in vaccine formulations, e.g., alum, and investigate whether its co-application might allow fewer injections than required today for the currently licensed vaccine formulations.

In order to understand the activity of dsRNA, we examined the innate response since this includes events that can improve the function of antigen presenting DCs and T cells. Surprisingly, we did not detect the expected increase of serum IFN-α shortly after injection of poly I:C or poly ICLC. This might be due to the s.c. route of injection. While i.v. injections of poly ICLC give rise to high serum interferon levels [30], the s.c. application of dsRNA may lead to a more protracted release from the site of injection and a delayed bioavailability. In mice, type I interferon induced by poly I:C has been shown to be essential for its adjuvant effect on humoral immunity and isotype switching [59], and it also seems essential for TLR3-mediated cross-priming of CD8^+^ T cells [60]–[62]. Likewise, type I interferon is critical for the CD8^+^ T cell expansion induced by TLR agonists in combination with CD40 [63]. Poly I:C and poly ICLC induce proliferation of CD8^+^ T cells, both have been shown to be effective as an adjuvant for the induction of specific CD8^+^ T cell responses in mice [64]–[66], and this effect partially depends on NK cells [67]. Thus, poly I:C, and most likely also poly ICLC, support the induction of CD8^+^ T cell responses, and the KLH-specific responses expressed by CD3^+^CD4^−^ T cells observed by us might reflect true CD8 responses.

In contrast to our inability to detect IFN-α in the serum in response to dsRNA, we did detect enhanced levels of CXCL10. These were sustained over 48 hours in animals injected with 0.5 mg/kg poly ICLC but decreased more rapidly in monkeys following injection of lower concentrations of poly ICLC, 0.5 mg/kg poly I:C, or a comparable dose (2 mg/animal) of poly I:C_12_U. This may reflect the reduced biostability of the nonstabilized poly I:C and poly I:C_12_U compared with that of poly ICLC as described before [29],[30]. CXCL10 is known for its activity to attract effector Th1 cells through interaction with its receptor CXCR3 at sites for the expression of Th1 immune responses [68], e.g., rejection of allografts or the inflammatory response upon mycobacterial infection [69],[70]. CXCL10 is also required for resistance to protozoan or viral pathogens [71],[72]. Studies in mice revealed additionally that CXCL10 is secreted early (e.g., earlier than CXCL9, which we did not detect at the same levels in the serum as CXCL10) [73], and stimulates T cell proliferation [74]. In fact, CXCL10-deficient mice have impaired T cell responses following primary immunization with exogeneous protein antigen indicating a role for CXCL10 in effector T cell generation [75]. Since CXCR3 also is induced early in CD4 T lymphocyte differentiation [76], the literature suggests an enhancing role for CXCL10 in both the expression and induction of Th1 immune responses. Notably, while we have previously detected an increase in serum CXCL10 after injection of CpG-A or CpG-B [14], these concentrations were around ten-fold lower than in animals injected with 0.5 mg/kg poly ICLC. Since both the two forms of CpGs and low doses of poly ICLC had only marginal adjuvant effects on the induction of cellular immunity, high and sustained serum levels of CXCL10 after injection of an adjuvant seem to be indicative of its ability to support the induction of cellular immune responses. Interestingly, the Th2-adjuvant alum considerably inhibits TLR-induced production of CXCL10 [77].

Expression of CXCL9 and CXCL10 was primarily in the T cell areas of the draining lymph node. Thus, DCs should be considered as a potential source of these chemokines, since they are abundant in this area of the lymph node. We show that monocyte-derived DCs produce CXCL10 upon activation with dsRNA, which suggests a direct role of these cells in the production of the pro-inflammatory chemokines and induction of cellular immune responses in our system. Monkey DCs express TLR3 (manuscript in preparation) and can be activated by poly I:C [58], so pattern recognition receptors on DCs likely contribute to the observed adjuvant effects of dsRNA for CD4^+^ T-cell proliferation. Synthetic dsRNA, however, may also target and activate other TLR3^+^ or TLR3^−^ leukocyte subsets. In vitro, it activates human NK cells [78],[79], γ/δ TCR^+^ T cells [80], CD8^+^ α/β TCR^+^ T cells [81], and also monocytes/macrophages, which are TLR3^−^ [82],[83]. These cells (or the corresponding cells in lymphoid tissues) could contribute to its adjuvant activity, e.g., through the secretion of pro-inflammatory cytokines and notably type I and II interferons. While it remains to be determined whether dsRNA can promote survival of primate CD4^+^ T cells as recently shown for murine cells [84], analyses of human blood leukocytes shortly after poly ICLC injection revealed increased percentages of CD4^+^ T cells, but also effects on the activity of NK cells and the frequency of HLA-DR^+^ cells [85]. Nevertheless, cells other than leukocytes including keratinocytes and neurons also can produce type I interferons and other pro-inflammatory cytokines upon stimulation with poly I:C [86],[87].

After injection of dsRNA, we observed a down-regulation of the homeostatic chemokine CCL21, which attracts CCR7^+^ cells, such as DCs and naïve T cells, to lymph nodes. In agreement with the advuvant effect of poly ICLC on the induction of HPV-specific Th1 immune responses shown in the present study, this process has recently been described for the early phase of the induction of Th1 but not Th2 immune responses in mice and is controlled by the production of IFN-γ [88]. This is mirrored by our findings using HPV16 capsomeres as viral protein antigen with relevance to the human system. Animals injected with HPV together with poly ICLC developed Th1 immune responses characterized by antigen-specific T cell proliferation and IFN-γ secretion in the absence of detectable IL-4 or IL-17 production.

In conclusion, dsRNA compounds induce the innate production of CXCL10 in the draining lymph nodes and high CXCL10 concentrations in the serum early after injection, and these compounds are effective adjuvants for the induction of adaptive pathogen-specific T cell and humoral immune responses.

## Materials and Methods

### Animals and immunizations

Healthy young adult male and female rhesus macaques (*Macaca mulatta*) housed at the German Primate Center (Deutsches Primatenzentrum, Göttingen, Germany) were used. The animals were antibody negative for simian T-lymphotropic virus type 1, simian D-type retrovirus, and simian immunodeficiency virus. All animal care operations were in compliance with the guidelines of the German Primate Center and approved by the local authorities. For immunizations and collection of blood samples animals were sedated with ketamine. For the removal of lymph nodes, a deeper anesthesia consisting of a mixture of xylazine, atropine, and ketamine was used. 200 µg endotoxin-free KLH (Calbiochem, San Diego, CA, USA) or 10 µg HPV 16 capsomeres alone or in combination with either poly I:C (Invivogen/Cayla, Toulouse, France), poly ICLC (Hiltonol, Oncovir, Washington, D.C.), poly I:C_12_U (Ampligen, Celldex Therapeutics, Bloomsbury, NJ, USA), or CpG-C (ODN 2396, generously provided by Coley Pharmaceutical Group, Wellesley, MA, USA) were administered bilaterally s.c. at doses indicated in the text at volumes between 1.0 and 2.0 ml, partially diluted in PBS, by injecting close to the inguinal lymph nodes. All animals remained well following the application of dsRNA plus antigen and no local signs of inflammation apart from transient lymph node swellings and mils hyperemia were observed at sites of injection.

Blood samples were drawn at 0, 1 or 6, 18 or 24, and 48 h after injections for measurements of serum cytokine and chemokine concentrations. To determine humoral and cellular immune responses additional blood samples were drawn at points in time indicated. Axillary lymph nodes were removed before the immunizations, and 18 h after the injections one draining lymph node from each immunized animal was removed. Lymph nodes were divided in two parts. One part was fixed in 4% neutral-buffered formalin overnight and embedded in paraffin. The other halves were embedded in tissue-freezing medium (Leica, Nussloch, Germany), snap-frozen in liquid nitrogen, and stored at −70°C until use.

### Purification of HPV16 L1 particles

HPV16 L1 capsomeres were produced using recombinant baculoviruses containing the mutated L1 (L1_2xCysM: C175A+C428A) as described previously [56]. In short, High Five insect cells (Invitrogen, Germany) were infected with recombinant baculoviruses and harvested by centrifugation. Proteins were extracted by sonification from cell pellets resupended in 20 ml of extraction buffer (5 mM MgCl2, 5 mM CaCl2, 1 M NaCl, 0.01% Triton ×100, 20 mM Hepes pH 7.4 and 1 mM PMSF). The cleared lysate was loaded on a two-step gradient consisting of (30% w/v) sucrose and CsCl (58% w/v), followed by a centrifugation at 96,500 g at 10°C for 3 h in a SW32 rotor (Beckman Ultracentrifuge). The interphase between the sucrose and CsCl and the complete CsCl layer were centrifuged again in Quickseal tubes (Beckman, USA) for 16–18 h at 20°C at 184,000 g in a Sorval TFT 65.13 rotor. Fractions of 1 ml fractions were collected and the L1 containing determined by antigen-capture ELISA and western blot analysis and the structure of the particles was characterized by electron microscopy [89]. The control antigen (mouse norovirus VP1 VLPs) were generated by the identical protocol. The VP1 clone was kindly provided by W. Nicklas, DKFZ Heidelberg.

### T cell assays

Standard proliferation assays were set up with 1×10^5^ PBMCs/well in 96-well round-bottom trays (Nunc) with KLH (100 µg/ml) in cell culture medium consisting of RPMI 1640, supplemented with 2 mM L-glutamine, penicillin (100 U/ml)-streptomycin (100 µg/ml), 10 mM HEPES (all GIBCO, Invitrogen, Karlsruhe, Germany), 50 µM 2-mercaptoethanol (Sigma), and 10% heat-inactivated FCS (Biochrom, Berlin, Germany). Controls included PBMCs in medium alone and PBMCs stimulated with 5 ng/ml staphylococcal enterotoxin B (SEB; Alexis Corp., Lausen, Switzerland). All conditions were set up in triplicates and cultures were incubated at 37°C and 5% CO_2_. Supernatants were harvested on day 2 and frozen at −80°C for analyses of cytokine concentrations. ^3^H-thymidine (1 µCi/well, NEN, Perkin Elmer, Boston, MA, USA) was added to the wells on day 3 (for SEB and medium alone) or day 5 (KLH and medium alone). Cells were harvested 24 h later onto glass fibre filter mats (ICN Biomedicals, Aurora, OH, USA), and incorporated ^3^H-thymidine was measured in a liquid scintillation counter. To facilitate the comparison of proliferative responses, SIs were calculated by dividing the mean counts per minute (cpm) of triplicates of antigen-containing wells by the mean cpm of triplicate wells with unstimulated PBMCs.

Additionally, CFSE (Invitrogen/Molecular Probes, Karlsruhe, Germany) assays were used to determine proliferation. PBMCs at 1×10^7^ cells/ml were stained with 0.25 µM CFSE in pre-warmed PBS for 15 min at 37°C, washed in medium, incubated in pre-warmed medium for another 30 min, and washed again. The cells were then adjusted to 1×10^6^ cells/ml and cultured in medium with or without SEB or KLH as described above or and incubated for 6 to 7 days. Alternatively, cells were incubated at 1.25 µg/ml with HPV16 VLPs or an unrelated control antigen, i.e., mouse norovirus VLPs similarly produced as the HPV antigen (A447), at the same dose. After 7 days cells were harvested and washed in PBS/5% FCS/0.05% sodium azide, stained with anti-CD3 PerCP- and anti-CD4 APC-conjugated mAbs, washed, and fixed. T cell proliferation was assessed as the percentage of CFSE^low^ cells, gating on live CD3^+^CD4^+^ or CD3^+^CD4^−^ cells (Figure 1A). Alternatively, cells were re-stimulated with eight pools of HPV16-specific, 15mer peptides (124 peptides, pool 1–4 with 16 peptides each, pool 5–8 with 15 peptides each), 2 µg/ml SEB, or medium alone in the presence of 1 µg/ml co-stimulatory mAbs CD28 and CD49d (BD Pharmingen) for 6 h, and Brefeldin A (Sigma) was added at a final concentration of 10 µg/ml for the last 4.5 h. Cells were then washed in PBS/5% FCS/0.05% sodium azide, stained with anti-CD3 PerCP- and anti-CD8 APC-conjugated mAbs, washed, fixed with 4% paraformaldehyde, and stained with PE-conjugated mAbs against IFN-γ after cell permeabilization with 0.5% saponin in PBS/5% FCS/0.05% sodium azide. T cell proliferation was assessed as the percentage of CFSE^low^ cells, gating on live CD3^+^CD8^+^ or CD3^+^CD8^−^ cells, and IFN-γ secretion was measured as the percentage of PE-stained, CFSE^low^ cells in the gated cell populations.

ELISPOT assays were preformed using commercially available reagents (Mabtech AB, Hamburg, Germany) as previously described [90]. Briefly, PBMCs were resuspended in culture medium and seeded at 1×10^5^ cells/well in 96-well plates (MAIP S4510, Millipore, Schwalbach, Germany), which had been coated with 1 µg/well of anti-human IFN-γ monoclonal antibody overnight at 4°C. For antigen stimulation, HPV16 L1 protein or control antigen (A447) was added at 1.25 µg/ml to the wells in triplicates. Positive and negative controls consisted of cells stimulated by SEB (1 µg/ml, Sigma) and cells kept in medium alone. After 20 h of incubation at 37°C in 5% CO_2_, cells were removed and biotinylated anti-human IFN-γ detector antibody was added (0.1 µg/well), followed by the addition of streptavidin-alkaline phosphatase conjugate at 1∶1000 in PBS/0.1% FBS. Spots were developed with NBT/BCIP solution (25 µg NBT and 15 µg BCIP in 0.1 M Tris–HCl pH 9.5 per well) for 30 min, the wells were washed with distilled water and air-dried, and spots were counted using a BIOSYS2000 ELISPOT reader. The counts were extrapolated to 10^6^ PBMCs. Average spot numbers of background responses (to A447) plus twice the standard deviation were considered positive responses.

### Measurement of antibody responses by ELISA

The presence of L1-specific IgG antibodies in plasma samples from immunized monkeys was determined by VLP-ELISA as described earlier [56]. Briefly, 96-well plastic plates were coated overnight at 4°C with VLP produced in baculovirus infected High Five insect cells and purified according to a previously published method [89]. After washing with PBS-T, plates were blocked with MPBS-T (5% skim milk in PBS- 0–05% Tween) for 1 hr at 37°C. Prediluted sera (in two-fold dilutions starting from 1∶50 to 1∶819,200) were added, and plates were incubated for 1 hr at 37°C. After washing, plates were incubated for 1 hr at 37°C with 1∶2000 diluted HRP-coupled antihuman IgG F(ab')_2_ secondary antibody (Dianova, Germany) in MPBS-T, TMB (3,3′,5,5 -tetramethylbenzidine) substrate solution (Sigma, Germany) was used as substrate. OD was measured in an ELISA reader at 450 nm after 10 min and 30 min incubation at room temperature. Nonspecific binding was determined by using the same dilutions on plates coated with extracts of High Five cells infected with wt baculovirus. IgG titers were expressed as the reciprocal of the highest dilution giving an absorbance above the cut off value (the average of the negative controls plus three times standard deviation).

### Pseudovirion-based neutralization assay [91]

Pseudovirions were prepared by transfecting 293TT cells (cultivated in DMEM containing 50 µg of hygromycin/ml) with a plasmid coding for the humanized HPV16 L1 and L2 genes, together with a plasmid containing the gene for secretable alkaline phosphatase (SEAP) under the control of the CMV promoter. For pseudovirion extraction, cells were harvested 3–4 days later by trypsination, washed once with PBS and resuspended in 1 ml PBS containing 1 mM CaCl_2_ and 5.6 mM MgCl_2_ per 5×10^7^ cells and lysed by 50 µl Brij58 (Sigma) in the presence of Benzonase (250 U/ml) for 5 min on ice. The cellular lysate was centrifuged after the addition of NaCl to a final concentration of 710 mM, and the cleared supernatant containing the pseudovirions was used for infection of 293TT cells. For this purpose, pseudovirions were diluted 1∶5000 in DMEM and preincubated with the sera (dilution from 1∶50 to 1∶100,000) for 15 min at room temperature. Pseudovirions were then added to the cells, followed by incubation at 37°C for 5 days. Detection of SEAP activity in cell culture supernatant was measured by using a commercial assay (Roche, Mannheim, Germany) according to the manufacturer's recommendations.

### Chemokine and cytokine secretion

Chemokine and cytokine concentrations in serum or plasma samples and cell culture supernatants were measured using ELISA kits for human CXCL10, CXCL9 (both R&D Systems, Wiesbaden, Germany), CCL3 (Antigenix America, Huntington, NY, USA), IFN-α (PBL, Brunswick, USA) [14], IL-17 (eBioscience, NatuTec, Frankfurt/Main, Germany) [57], and human TNF as well as monkey IFN-γ, IL-4, and IL-12p40 (all U-Cytech, Utrecht, The Netherlands).

### Immunohistochemistry

Cryostat sections were cut, fixed in acetone for 30 min and incubated with monoclonal antibodies against human CD1a (dilution: 1∶100; Medac, Hamburg, Germany), CD83 (dilution: 1∶100) or CD208 (1∶70; both Immunotech, Hamburg Germany). Antibody binding was visualized by the alkaline phosphatase anti-alkaline phosphatase method using New Fuchsin as chromogen. The sections were counterstained with hemalaun and mounted. The numbers of DCs were quantified with a Zeiss AxioImager M1 microscope (Carl Zeiss, Jena, Germany). Using a 40× objective, a standard area was set (unit area). Ten non-overlapping unit areas were selected. The positive cells were counted using AxioVision (Release 4.6) software (Zeiss). The values were averaged to represent the numbers of positive cells per unit area. Due to inadequate immunohistochemical staining the draining lymph node from animal number 13408 was omitted from the examination.

Immunohistochemistry on paraffin sections was performed as previously described [92]. The following antibodies diluted in antibody diluent (S3022, DAKO, Glostrup, Denmark) were used: mouse anti-CXCL10 (MAB266, R&D Systems, 1 µg/ml), goat anti-CXCL9 (AF392, R&D Systems, 1 µg/ml), and goat anti-CCL21 (AF366, R&D Systems, 1 µg/ml). After over night incubation, sections were washed and incubated with rabbit anti-mouse (E0413, DAKO) or rabbit anti-goat (E0466, DAKO,) biotinylated antibodies followed by streptavidin-alkalyne phosphatase complex (K0391, DAKO), following the manufacturer's instructions. Positive cells were detected using New Fuchsin (K0698, DAKO) as substrate, and tissue sections counterstained with Meyer's Haematoxylin (1.09249, Merck, Zug, Switzerland).

### In situ hybridization

^35^S-labeled sense and antisense CXCL9, CXCL10, and CCL21 mRNA probes, 411 bp in length corresponding to position 26 to 437 of the CXCL9 sequence (NM_002416), 372 bp corresponding to position 28 to 400 of the CXCL10 sequence (NM_001565), and 367 bp corresponding to position 27 to 394 of the CCL21 sequence (NM_002989), respectively, were generated by *in vitro* transcription (Roche Molecular Biochemicals, Indianapolis, IN). Tissue sections were dewaxed, rehydrated in graded ethanol solutions, and subjected to *in situ* hybridization, according to a previously described method [93]. Finally, the sections were dipped in photo emulsion NTB-2 (Kodak, Rochester, NY) and exposed in complete darkness for 2 to 4 weeks at 4°C. Development and fixation were performed according to the instructions provided by Kodak, and counterstaining was done with haematoxylin.

### Generation of monocyte-derived DCs

Rhesus macaque monocyte-derived DCs were generated from heparinized peripheral blood as previously described [57]. CD14^+^ monocytes were magnetically separated (Miltenyi Biotec, Bergisch-Gladbach, Germany) and cultured at 1.5–2×10^6^ cells/3 ml in RPMI 1640, supplemented with 5% human AB serum (PAN Biotech, Aidenbach, Germany), human rGM-CSF (1000 U/ml, sargramostim, Leukine, Berlex, Richmond, CA, USA), human rIL-4 (100 U/ml, R&D Systems, Wiesbaden-Nordenstadt, Germany, and L-glutamine, 2-mercaptoethanol, HEPES, and penicillin-streptomycin as described under T cell assays. At day 6, DCs at 1×10^5^/well were stimulated for 48 h with 50 or 200 µg/ml poly ICLC in 96-well round bottom plates. Supernatants were harvested for analysis of cytokine and chemokine secretion.

### Statistical analysis

Data are expressed as means±standard error of the mean (SEM), standard deviation (SD), or median, where appropriate. Statistical significance of differences was determined by Student's t-test or Mann Whitney U-test. Differences were considered statistically significant for p<0.05.

## Supporting Information

Figure S1 Kinetics of proliferative responses in PBMCs after immunization with KLH and dsRNA. Rhesus macaques were immunized with KLH plus 0.5 mg/kg poly ICLC (A), KLH plus 0.5 mg/kg poly I:C (B), or KLH alone (C), and cellular immune responses were determined in proliferation assays. PBMCs were stimulated for 5 days with KLH (100 µg/ml), and 3H-thymidine was added for another 24 hours before measuring its incorporation. Stimulation indices were calculated for results of the individual animals (marked by the four- or five-digit numbers) by dividing the mean cpm of triplicates of antigen-containing wells by the mean cpm of triplicate wells with unstimulated PBMCs.
(0.73 MB TIF)

Figure S2 The adjuvant effect of dsRNA on KLH-specific proliferative immune responses is dose-dependent. Rhesus macaques were immunized with KLH (200 µg) plus poly ICLC at 0.5 mg/kg body weight (A) or 0.1 mg/kg (B) at weeks 0 and 14 (arrows), and KLH-specific proliferation of CD3^+^CD4^+^ T cells (solid lines) or CD3^+^CD4^−^ T cells (dotted lines) was assessed in CFSE dilution assays, incubated for 7 days. KLH-specific proliferation was measured as the proportion of CFSElow cells, gating on CD3-, CD4-double positive or CD3-positive, CD4-negative cells, respectively. Background proliferation in medium alone was subtracted from proliferation of KLH-stimulated PBMCs. The five-digit numbers are monkey designations.
(0.74 MB TIF)

Figure S3 Correlation between antibody titers measured by ELISA and neutralization assays. Titers in serum samples collected 12 weeks after the first immunization with HPV16 capsomeres (10 µg/animal) alone or together with 2 mg of poly ICLC or CpG-C are shown for the individual animals. Neutralization titers are given as reciprocal of the highest dilution used in this experiment yielding ≥50% neutralizing activity. Determination of ELISA titers is described in Materials and Methods.
(0.76 MB TIF)

Figure S4 DC numbers in draining lymph nodes 18 hours after immunization of KLH plus poly ICLC. Numbers of CD1a, CD83, and CD208 positive cells per unit area were determined by immunohistochemistry in lymph node sections before and 18 hours after immunization with KLH plus poly ICLC at 0.5 mg/kg body weight (filled symbols) or 0.1 mg/kg (open symbols).
(0.37 MB TIF)

Table S1 Maximum proliferative responses (mean cpm of wells in triplicates) after immunization of rhesus macaques with KLH (200 µg) alone or together with poly I:C or poly ICLC (0.5 mg/kg body weight).
(0.02 MB DOC)

Table S2 Individual titers of L1-binding antibodies after immunization with HPV16 capsomeres (10 µg) alone or together with poly ICLC or CpG-C (2 mg/animal).
(0.04 MB DOC)
