# Supplementary figures and images for: Synthetic Double-Stranded RNAs Are Adjuvants for the Induction of T Helper 1 and Humoral Immune Responses to Human Papillomavirus in Rhesus Macaques
Source: PLoS Pathog. 2009 Apr 10;5(4):e1000373. doi: 10.1371/journal.ppat.1000373 (PMC2660151; doi:10.1371/journal.ppat.1000373)

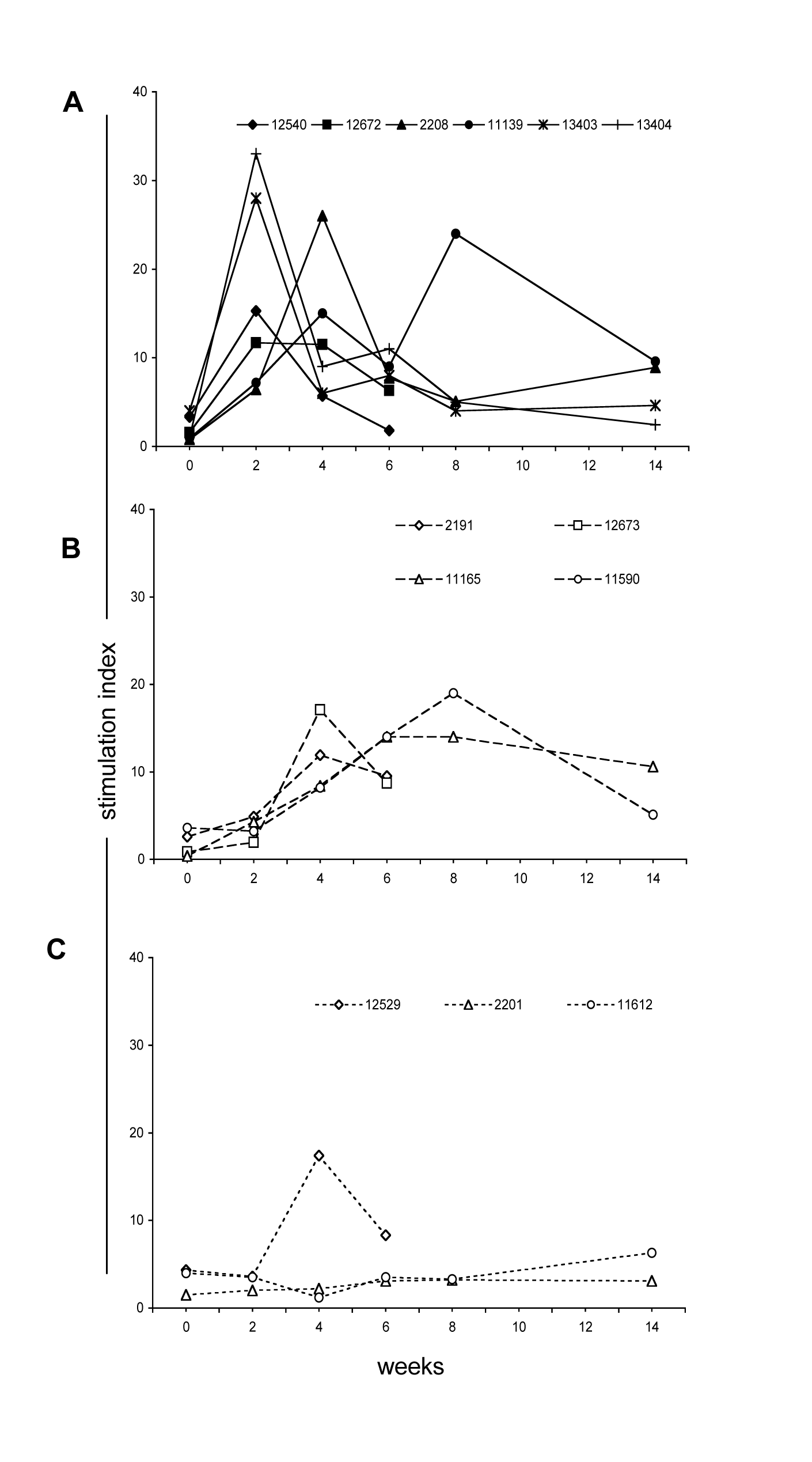

Supplement: Figure S1 — Kinetics of proliferative responses in PBMCs after immunization with KLH and dsRNA. Rhesus macaques were immunized with KLH plus 0.5 mg/kg poly ICLC (A), KLH plus 0.5 mg/kg poly I:C (B), or KLH alone (C), and cellular immune responses were determined in proliferation assays. PBMCs were stimulated for 5 days with KLH (100 µg/ml), and 3H-thymidine was added for another 24 hours before measuring its incorporation. Stimulation indices were calculated for results of the individual animals (marked by the four- or five-digit numbers) by dividing the mean cpm of triplicates of antigen-containing wells by the mean cpm of triplicate wells with unstimulated PBMCs. (0.73 MB TIF) [file ppat.1000373.s001.tif]

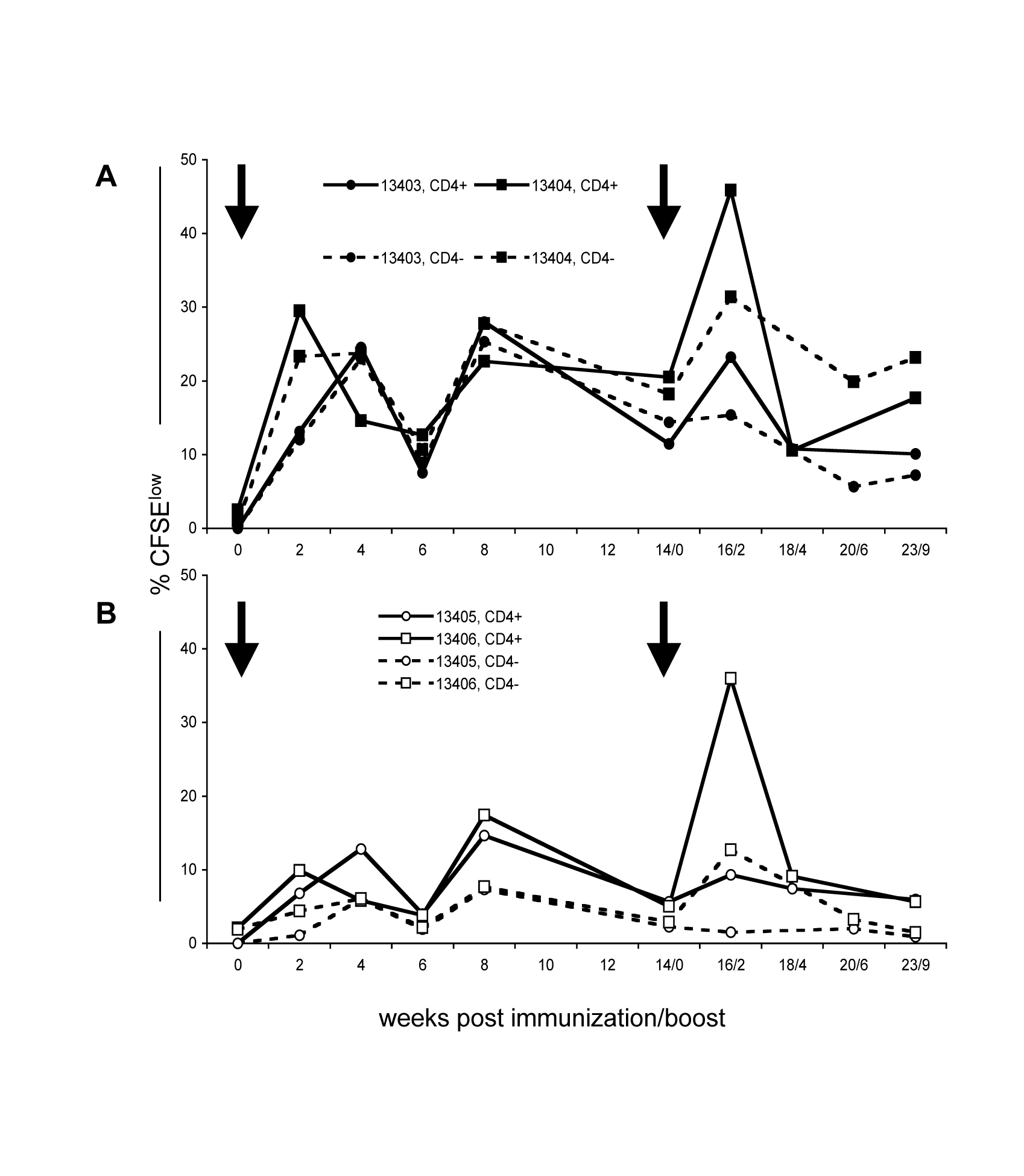

Supplement: Figure S2 — The adjuvant effect of dsRNA on KLH-specific proliferative immune responses is dose-dependent. Rhesus macaques were immunized with KLH (200 µg) plus poly ICLC at 0.5 mg/kg body weight (A) or 0.1 mg/kg (B) at weeks 0 and 14 (arrows), and KLH-specific proliferation of CD3+CD4+ T cells (solid lines) or CD3+CD4− T cells (dotted lines) was assessed in CFSE dilution assays, incubated for 7 days. KLH-specific proliferation was measured as the proportion of CFSElow cells, gating on CD3-, CD4-double positive or CD3-positive, CD4-negative cells, respectively. Background proliferation in medium alone was subtracted from proliferation of KLH-stimulated PBMCs. The five-digit numbers are monkey designations. (0.74 MB TIF) [file ppat.1000373.s002.tif]

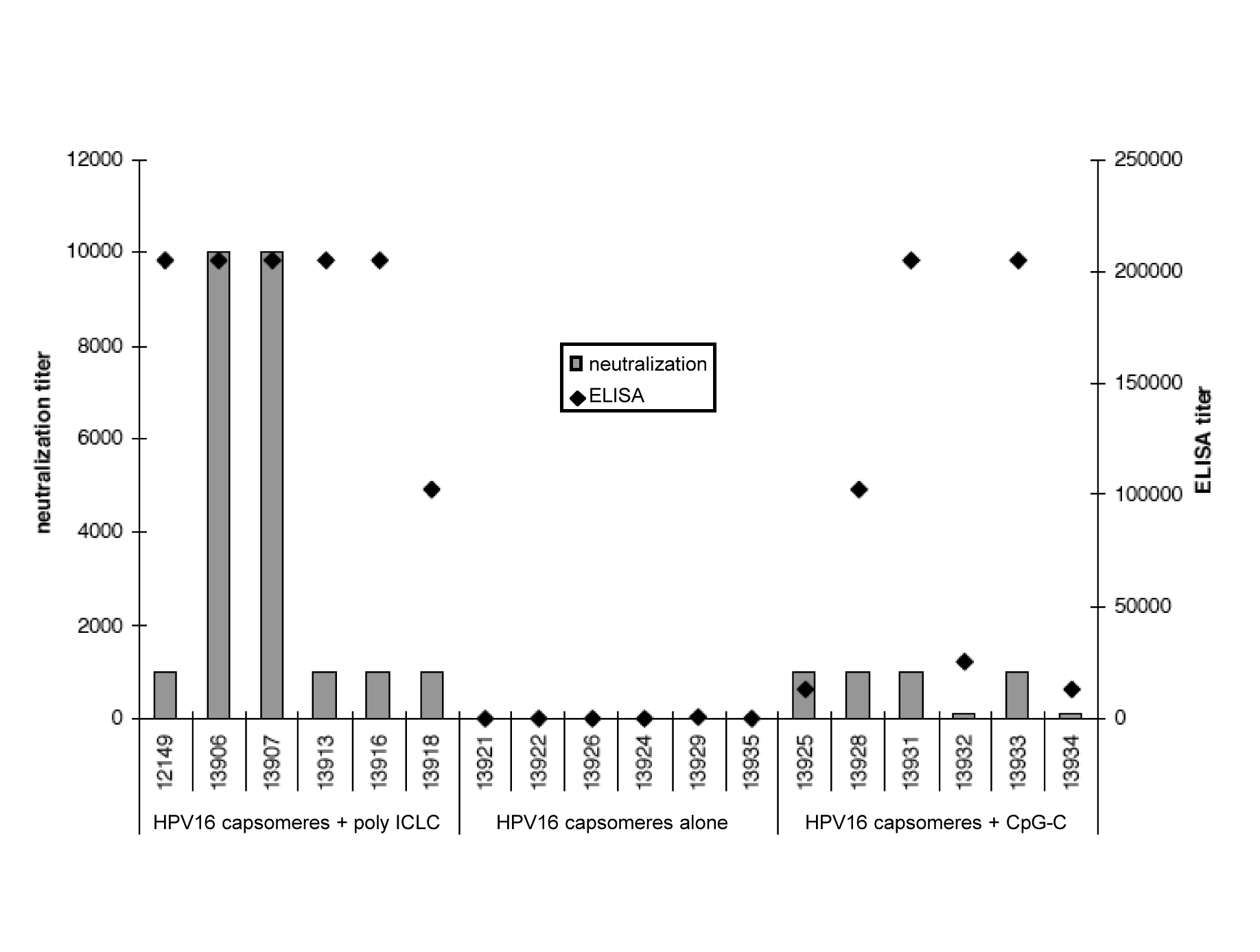

Supplement: Figure S3 — Correlation between antibody titers measured by ELISA and neutralization assays. Titers in serum samples collected 12 weeks after the first immunization with HPV16 capsomeres (10 µg/animal) alone or together with 2 mg of poly ICLC or CpG-C are shown for the individual animals. Neutralization titers are given as reciprocal of the highest dilution used in this experiment yielding ≥50% neutralizing activity. Determination of ELISA titers is described in Materials and Methods. (0.76 MB TIF) [file ppat.1000373.s003.tif]

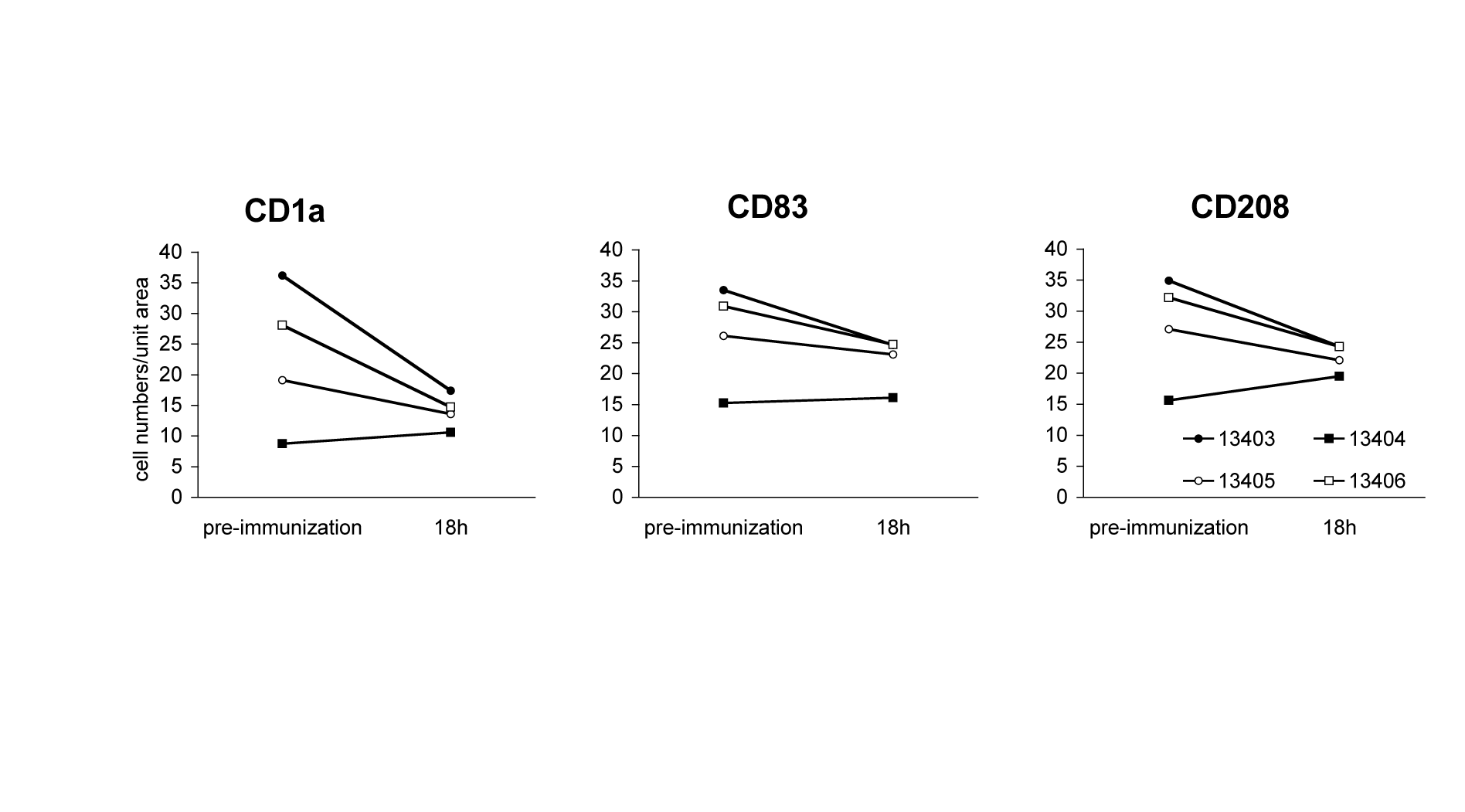

Supplement: Figure S4 — DC numbers in draining lymph nodes 18 hours after immunization of KLH plus poly ICLC. Numbers of CD1a, CD83, and CD208 positive cells per unit area were determined by immunohistochemistry in lymph node sections before and 18 hours after immunization with KLH plus poly ICLC at 0.5 mg/kg body weight (filled symbols) or 0.1 mg/kg (open symbols). (0.37 MB TIF) [file ppat.1000373.s004.tif]
