# Supplementary material for: Synthetic Double-Stranded RNAs Are Adjuvants for the Induction of T Helper 1 and Humoral Immune Responses to Human Papillomavirus in Rhesus Macaques
Source: PLoS Pathog. 2009 Apr 10;5(4):e1000373. doi: 10.1371/journal.ppat.1000373 (PMC2660151; doi:10.1371/journal.ppat.1000373)
Supplement: Table S1 — Maximum proliferative responses (mean cpm of wells in triplicates) after immunization of rhesus macaques with KLH (200 µg) alone or together with poly I:C or poly ICLC (0.5 mg/kg body weight). (0.02 MB DOC) [file ppat.1000373.s005.doc]

**Table S1.** Maximum proliferative responses (mean cpm of wells in triplicates) after immunization of rhesus macaques with KLH (200 μg) alone or together with poly I:C or poly ICLC (0.5 mg/kg body weight).

| Immunization | Animals | KLH* | Medium |
| --- | --- | --- | --- |
| KLH alone | 12529 | 30238 | 1,741 |
|  | 2201 | 1251 | 391 |
|  | 11612 | 9979 | 1,583 |
| KLH + poly I:C | 2191 | 50288 | 4,238 |
|  | 12673 | 31678 | 1,854 |
|  | 11165 | 6507 | 459 |
|  | 11590 | 18325 | 1,068 |
| KLH + poly ICLC | 12540 | 44331 | 2,897 |
|  | 12672 | 20697 | 1,765 |
|  | 2208 | 29721 | 1,150 |
|  | 11139 | 30974 | 1,287 |
|  | 13403 | 17757 | 644 |
|  | 13404 | 12527 | 378 |

* 10^5^ PBMCs stimulated with KLH (100 µg/ml) or kept in medium, all conditions set up in triplicates
