# Supplementary material for: Synthetic Double-Stranded RNAs Are Adjuvants for the Induction of T Helper 1 and Humoral Immune Responses to Human Papillomavirus in Rhesus Macaques
Source: PLoS Pathog. 2009 Apr 10;5(4):e1000373. doi: 10.1371/journal.ppat.1000373 (PMC2660151; doi:10.1371/journal.ppat.1000373)
Supplement: Table S2 — Individual titers of L1-binding antibodies after immunization with HPV16 capsomeres (10 µg) alone or together with poly ICLC or CpG-C (2 mg/animal). (0.04 MB DOC) [file ppat.1000373.s006.doc]

**Table S2.** Individual titers of L1-binding antibodies after immunization with HPV16 capsomeres (10 μg) alone or together with poly ICLC or CpG-C (2 mg/animal).

|  |  | Time post first immunization | | | | |
| --- | --- | --- | --- | --- | --- | --- |
| Immunization | Animals | pre | 4 | 8 | 10 | 12 |
| HPV16 capsomeres + poly ICLC | 12149 | 0 | 1600 | 3200 | 204800 | 204800 |
|  | 13906 | 0 | 1600 | 6400 | 409600 | 204800 |
|  | 13907 | 0 | 3200 | 12800 | 204800 | 204800 |
|  | 13913 | 0 | 800 | 3200 | 102400 | 204800 |
|  | 13916 | 0 | 800 | 3200 | 204800 | 204800 |
|  | 13918 | 0 | 400 | 6400 | 102400 | 102400 |
| HPV16 capsomeres + CpG-C | 13925 | 0 | 200 | 400 | 25600 | 12800 |
|  | 13928 | 0 | 400 | 800 | 51200 | 102400 |
|  | 13931 | 0 | 800 | 1600 | 102400 | 204800 |
|  | 13932 | 0 | 100 | 200 | 12800 | 25600 |
|  | 13933 | 0 | 800 | 3200 | 204800 | 204800 |
|  | 13934 | 0 | 100 | 200 | 25600 | 12800 |
| HPV16 capsomeres alone | 13921 | 0 | 0 | 0 | 400 | 100 |
|  | 13922 | 0 | 0 | 0 | 200 | 0 |
|  | 13924 | 0 | 50 | 200 | 400 | 100 |
|  | 13926 | 0 | 0 | 0 | 200 | 0 |
|  | 13929 | 0 | 0 | 100 | 800 | 400 |
|  | 13935 | 0 | 0 | 0 | 400 | 0 |
